# Is the Combination of Immunotherapy and Radiotherapy in Non-small Cell Lung Cancer a Feasible and Effective Approach?

**DOI:** 10.3389/fmed.2019.00244

**Published:** 2019-11-07

**Authors:** Mathieu Spaas, Yolande Lievens

**Affiliations:** Department of Radiation Oncology, Ghent University Hospital and Ghent University, Ghent, Belgium

**Keywords:** non-small-cell lung cancer, immunotherapy, checkpoint inhibitor, stereotactic body radiation therapy, radiotherapy

## Abstract

For many years, conventional oncologic treatments such as surgery, chemotherapy, and radiotherapy (RT) have dominated the field of non-small-cell lung cancer (NSCLC). The recent introduction of immunotherapy (IT) in clinical practice, especially strategies targeting negative regulators of the immune system, so-called immune checkpoint inhibitors, has led to a paradigm shift in lung cancer as in many other solid tumors. Although antibodies against programmed death protein-1 (PD-1) and programmed death ligand-1 (PD-L1) are currently on the forefront of the immuno-oncology field, the first efforts to eradicate cancer by exploiting the host's immune system date back to several decades ago. Even then, researchers aimed to explore the addition of RT to IT strategies in NSCLC patients, attributing its potential benefit to local control of target lesions through direct and indirect DNA damage in cancer cells. However, recent pre-clinical and clinical data have shown RT may also modify antitumor immune responses through induction of immunogenic cell death and reprogramming of the tumor microenvironment. This has led many to reexamine RT as a partner therapy to immuno-oncology treatments and investigate their potential synergy in an exponentially growing number of clinical trials. Herein, the authors review the rationale of combining IT and RT across all NSCLC disease stages and summarize both historical and current clinical evidence surrounding these combination strategies. Furthermore, an overview is provided of active clinical trials exploring the IT-RT concept in different settings of NSCLC.

## Introduction

Radiotherapy (RT) has earned its place as one of three main pillars in non-small cell lung cancer (NSCLC) treatment, alongside surgery and systemic agents. Traditionally considered as a means of achieving local tumor control through induction of irreversible deoxyribonucleic acid (DNA) damage in irradiated tumor cells, RT is used in routine clinical practice across all NSCLC disease stages, whether with curative or palliative intent (1). However, since as early as 1953, reports have been published describing tumor regression outside the radiation fields (2). This so-called “abscopal effect” has more recently been postulated to be the result of a RT-induced antitumor immune response. With the advent of modern immunotherapy (IT), the potential for immune activation by RT has become even more relevant. Indeed, mounting pre-clinical and clinical evidence suggests a potential synergy between RT and IT, creating opportunities for combining these two treatment strategies, in NSCLC as in many other tumor types. In order to harness these synergistic effects however, it is important to understand the underlying mechanisms in which key factors such as the type of IT used, the irradiated volume, as well as timing, dose and fractionation of RT play a crucial role. Herein, we review the rationale for combining IT and RT across different NSCLC disease stages and summarize the current clinical evidence surrounding these novel treatment approaches. Furthermore, an overview is provided of active clinical trials exploring the IT-RT concept in different settings of NSCLC.

For this review, we conducted a search of PubMed, Embase and Web of Science for original research, review articles and meta-analyses relevant to the combination of RT and IT in NSCLC from inception until March 2019, yielding a total reference count of 708. After removal of duplicates, 394 abstracts were screened by one reviewer, of which 62 qualified for full text screening. In case of multiple publications reporting on the same study population, manuscripts with the longest follow-up were selected for inclusion. The cited and citing references of the included studies were checked for additional relevant publications. Finally, a total of 42 published original research papers and abstracts were included and their results will be discussed below. Furthermore, using the search terms “radiotherapy,” “immunotherapy,” “immune,” “vaccine,” and “checkpoint,” the international clinical study database Clinicaltrials.gov was queried for currently active trials in the area of NSCLC combining both treatment modalities.

## Immunologic Effects of Radiotherapy

It has been over a decade since new insights into the complex interplay between cancer and the host's immune system, known as the cancer immunoediting hypothesis (3), have revolutionized our understanding and approach to this disease. Besides sparking interest in the development of novel immunotherapeutic drugs targeting different aspects of the so-called cancer-immunity cycle (4), it also prompted researchers to reassess the role of conventional oncological therapies—most notably RT—in anticancer immunity.

### *In-situ* Vaccination

**Figure 1:**
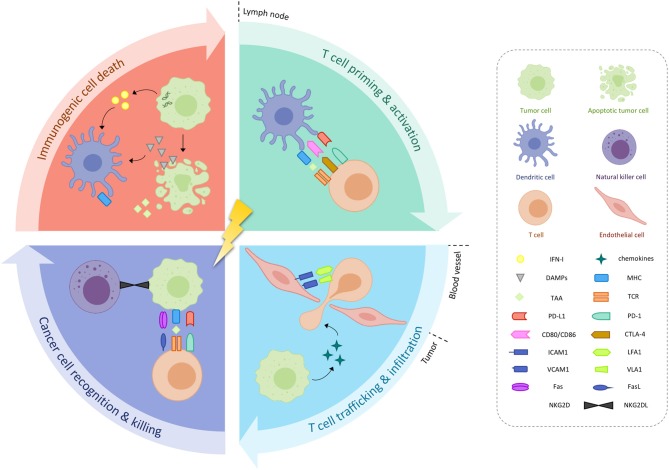
Immunological effects or radiotherapy. Radiotherapy may induce immunogenic cancer cell death, characterized by increased expression of danger-associated molecular patterns (DAMPs) and type I interferon (IFN-I), in turn causing the release of tumor-associated antigens (TAAs). Activated dendritic cells (DCs) will present these TAAs to T-cells located in the tumor-draining lymph node, which also carry inhibitory receptors programmed death protein 1 (PD-1) and cytotoxic T-lymphocyte-associated antigen 4 (CTLA-4) on their cell surface. T-cell homing back to the tumor microenvironment is aided by radiation-induced chemokines, as well as upregulation of intercellular (ICAM) and vascular cell adhesion molecules (VCAM) on endothelial cells. Increased expression of major histocompatibility complex (MHC), Fas and natural killer group 2, member D (NKG2D) by residual irradiated tumor cells facilitates their destruction. CD, cluster of differentiation; L, ligand; LFA1, lymphocyte function associated antigen 1; PD-L1, programmed death ligand-1; TCR, T-cell receptor; VLA1, integrin alpha 1.

The principal mechanism of action of ionizing radiation is the induction of irreparable DNA damage in tumor cells—either directly or indirectly through free radicals. Under the right circumstances, radiation-damaged tumor cells may in turn undergo a phenomenon called “immunogenic cell death,” whereby an increased expression of calreticulin facilitates their phagocytosis by dendritic cells (DCs) and promotes the secretion of pro-inflammatory cytokines (Figure 1) (5). In addition, radiation-induced DNA damage leads to the accumulation of cytosolic DNA, which stimulates the production of type I interferons (IFN-I) through cyclic guanosine monophosphate-adenosine monophosphate synthase (cGAS)/stimulator of IFN genes (STING) nucleic acid-sensing pathways (6–8). RT also triggers the release of several other danger-associated molecular patterns, including adenosine triphosphate and high mobility group box 1, which together with IFN-I, prompt DC recruitment and activation (5, 9). After subsequently migrating to the tumor-draining lymph node, DCs will present tumor-associated antigen (TAA) to cluster of differentiation 8 positive (CD8+) T-cells so that cross-priming and activation of these cytotoxic T-cells can occur (10, 11). T-cell trafficking back to the tumor microenvironment is aided by radiation-induced chemokines such as C-X-C chemokine ligand 16 (CXCL16) by the tumor and intercellular (ICAM) and vascular cell adhesion molecules expression by the endothelial cells (12, 13). There, cytotoxic T lymphocytes will meet residual irradiated tumor cells that show increased expression of major histocompatibility complex class I (MHC-I), Fas and natural killer group 2, member D ligands, thus rendering them more sensitive to cell killing (14–16). In theory, these TAA-specific T-cells could also home to cancerous lesions outside of the radiation field, thereby leading to abscopal responses.

### The Role of RT Dose

It has been stipulated that RT in conventional dose-fractionation regimens [i.e., 1.8–2 Gray (Gy) per fraction] may elicit profound immunosuppressive responses in tumors. Such effects include recruitment of notoriously pro-tumorigenic myeloid-derived suppressor cells (MDSCs) and M2 tumor-associated macrophages (TAM), as well as a preferential increase of the regulatory T-cell (Treg) population, either independently, due to their intrinsic radioresistance or as a consequence of RT-induced upregulation of transforming growth factor beta (3, 17–20). Conversely, 2 Gy daily RT fractions may also have the potential to boost antitumor immune responses through *in-situ* vaccination, as demonstrated by the detection of TAA-specific CD8+ T-cells in the circulation of colorectal (21) and prostate cancer patients (22) receiving standard (chemo)radiation. Nevertheless, in pre-clinical experiments comparing immunologic effects of conventional RT doses to those of hypofractionated regimens, more specifically if ≥6 Gy per fraction is being delivered, or even single high-dose radiation, profound differences are observed (9). For example, Reits et al. showed that the expression of MHC-I and associated tumor peptides was higher with increasing RT doses (16). Other reports demonstrate that higher doses may lead to greater upregulation of other stimulatory immune signals such as Fas and ICAM, as well as enhanced tumor-specific CD8+ T-cell infiltration (23, 24).

Interestingly, the enhanced immunogenicity of increasing radiation doses does not seem to extend beyond a certain dose range (24). This could be explained in part by the induction of DNA exonuclease Trex1 at doses larger than 18–20 Gy per fraction, which degrades cytosolic DNA, thus preventing activation of the cGAS/STING pathway and thereby abrogating a potential antitumor immune response (8).

### The Role of RT Timing and Sequencing

Immunologic effects of RT may not only be sensitive to variations in dose and fractionation, they also appear to be time-dependent. For instance, *in vitro* data demonstrate an increase in the MHC-I-associated peptide pool after approximately 8 h and up to 11 days or more following high doses of radiation (16). Kinetics of RT-induced intratumoral immune cell activation were also studied *in vivo*, particularly in cervical cancer patients treated with conventionally fractionated RT and concurrent chemotherapy (25). Repeated cervical brushings showed an enrichment of activated DCs at the tumor site during the first week of chemoradiation, potentially corresponding to treatment-induced antigen presentation. Meanwhile, the proportion of activated and proliferating T-cells experienced an initial decline in week 1, but quickly recovered and even increased in weeks 3–5, supporting the *in-situ* vaccination hypothesis. Interestingly, changes in the populations and/or activation status of myeloid and T-cells appeared to be more pronounced at the tumor site in comparison to peripheral blood samples. Findings such as these provide important insights into immune dynamic changes in the tumor microenvironment and could therefore further the development of rational IT-RT combination strategies, especially regarding optimal treatment sequencing.

The antitumor effects of antigen-specific immunotherapy (ASI), for instance, rely on the generation of a potent cellular immune response against a specific TAA. A prime example of this is tecemotide, a peptide vaccine containing a liposomal formulation of tumor-associated mucin 1 (MUC1), which is designed to elicit a MUC1-specific T-cell proliferative response. As the initial depletion of effector cell types from the tumor microenvironment caused by repeated moderate doses of RT could abrogate any previously induced immune infiltrate, ASI such as tecemotide may prove most useful when administered after RT, serving as a booster for the immune cells generated by *in-situ* vaccination. Moreover, the RT-induced expression of chemotactic signals such as CXCL16 may facilitate homing of any TAA-specific T-cells to the irradiated lesion.

Similarly, RT may be used to prime tumors in order to render them more susceptible to adoptive IT, such as lymphokine-activated killer cell (LAK), DC, cytokine-induced killer cell (CIK), tumor-infiltrating lymphocyte (TIL) and chimeric antigen receptor (CAR) T-cell therapy. Important work by Klug et al. demonstrated in murine as well as xenotransplanted and human primary tumors that even single low doses of RT ( ≤ 2 Gy) may be capable of reprogramming the tumor microenvironment—in particular polarizing TAMs to an M1 phenotype—causing inducible nitric oxide synthase-mediated vascular normalization, and thus facilitating tumor homing of transferred T-cells (26). In addition, RT may enhance cytotoxicity of CIKs through upregulation of signaling pathways required for their antitumor activity, including NK-cell receptor ligands and Fas. In turn, CIKs are known to secrete proinflammatory cytokines such as tumor necrosis factor alpha and interferon gamma (IFNɤ), thereby enriching local immune responses (27). With regards to CAR T-cell therapy, De Selm et al. demonstrated increased efficacy after previous exposure of tumors to low-dose RT in their orthotopic pancreatic cancer model (28).

Of all IT-RT combinations, treatment regimens integrating RT and immune checkpoint blockade (ICB) have garnered the most interest in recent years. Simply put, the rationale of a potential synergy between both treatments is that the inhibition of immune checkpoints, such as cytotoxic T lymphocyte antigen 4 (CTLA-4), programmed cell death protein 1 (PD-1) and programmed cell death 1 ligand 1 (PD-L1), liberates T-cells from immunosuppression, thereby increasing the *in-situ* vaccination effect of RT. Additional benefits of ICB in the context of RT include: depletion of intratumoral Treg cells (anti-CTLA-4), counteracting RT-induced T-cell exhaustion through upregulation of PD-L1 (anti-PD-(L)1) and a reduction of MDSC populations in the tumor microenvironment (anti-PD-(L)1) (29). RT can also lead to upregulation of PD-L1 expression in tumors, which has been shown to be mediated by IFNɤ and may be an important mechanism of radioresistance (30). Altogether, these findings suggest concurrent administration of ICB with RT would be optimal in order to exploit their full synergistic potential. Nevertheless, encouraging results have been achieved with sequential treatment regimens as well, in particular with the adjuvant use of checkpoint inhibitors to CRT. In these types of treatment schedules, timing may play an even greater role, as in the PACIFIC trial, where multivariate analysis suggested that initiation of anti-PD-L1 treatment before 2 weeks after completion of chemoradiation was correlated with better overall survival (OS) in comparison to initiation after 14 days or more (31). Nevertheless, further analysis showed that durvalumab initiated after longer time intervals still provided clinical benefit in terms of progression-free survival (PFS) and time to distant metastasis (32).

Less is known about the underlying mechanisms of interaction between RT and non-specific immunomodulatory drugs, such as levamisole, or so-called active immunotherapy (i.e., Bacillus Calmette-Guérin, BCG), making it more difficult to theorize their optimal sequencing.

### The Role of RT Target Volume and Organs at Risk

As illustrated above, lymphocytes play a crucial role in antitumor immunity. Unfortunately, one of the major drawbacks of conventional oncological treatments is their tendency to cause systemic lymphopenia, which is increasingly being recognized to profoundly influence outcome. Indeed, several reports have shown radiation-induced lymphopenia (RIL) to be an independent predictor for poor survival in solid tumors (33, 34). More specifically in NSCLC, besides the obvious consideration of whether or not a patient is amenable to receive concurrent chemotherapy, the definition of RT target volumes seems to be a determining factor in the incidence of RIL. First, the sheer size of the radiation field has a direct impact on lymphocyte nadirs, as was demonstrated in a retrospective study by Tang et al. (35). This effect could be explained by taking into consideration unintentional RT doses to sites of lymphopoiesis (bone marrow) and lymphocyte storage (spleen, lymph nodes), as well as the amount of circulating immune cells passing through the radiation field (36, 37). The latter is of particular importance in thoracic RT, where organs at risk (OARs) characterized by high blood flow such as the heart and lungs reside, thus exposing a large proportion of circulating lymphocytes to radiation (33). Due to their intrinsic radiosensitivity to doses even below 1 Gy (38) large volumes irradiated to relatively low doses (i.e., the low-dose bath), inherent even to modern photon RT techniques such as intensity-modulated radiotherapy, may significantly impact the incidence of RIL. In theory, more conformal (e.g., proton therapy) and faster (e.g., higher dose rate) dose delivery could aid in limiting this exposure.

In addition to the direct hit of lymphocytes by RT, pre-clinical data suggest irradiation of the draining lymph nodes may also attenuate adaptive immune responses through altered intratumoral chemokine expression and CD8+ T-cell trafficking as compared to RT to the primary tumor alone (39). This in turn adversely affected treatment outcome when RT was combined with ICB.

Adding to the list of OARs to consider, Price et al. demonstrated irradiating major areas of skin may also have detrimental effects on antitumor immune responses. Their experiments showed RT mobilizes Langerhans cells, a DC subset present in the epidermis, which may subsequently migrate to the draining lymph node where they cause an accumulation of Treg cells (40).

On the other end of the benefit-risk balance of IT-RT strategies stands the possibility of increased toxicity of combined treatment. RT-induced immune-cell infiltration and subsequent inflammation-associated normal tissue responses underlying the occurrence of radiation-induced lung injury (i.e., radiation pneumonitis and fibrosis) warrant further inquiry into a potential interaction with immunotherapy (41). Most notably, it has been postulated that combining RT with ICB may pose a particular risk, as these drugs alone are known to cause severe, even potentially life-threatening, pneumonitis in a small subset of patients. Reports of high-grade lung toxicities with concurrent RT and anti-PD-(L)1 caution the use of such treatment schedules outside of clinical trials (42). Even so, higher rates of pneumonitis may be expected in a non-trial-enrolled population, as is the case with ICB monotherapy (43). Regardless of the setting, all available measures should be taken to minimize the incidence of RT-related pulmonary toxicity, such as careful patient and tumor selection, as well as optimal treatment planning and delivery.

## Combining radiotherapy and immunotherapy in NSCLC

### Locally-Advanced NSCLC

Approximately one in four new NSCLC cases are diagnosed with locally-advanced (LA-NSCLC) disease (44). In this setting, RT may be offered as an adjunct to surgery in operable patients, be it in the preoperative setting or after incomplete resection, but will most frequently be used as definitive treatment combined with chemotherapy (concurrently of sequentially) in stage IIIB or (unresectable) stage IIIA disease. When combined with platinum-based doublet chemotherapy, RT doses ranging between 60 and 66 Gy in 2 Gy daily fractions over 6–7 weeks are advocated (1). Despite many historical efforts to optimize treatment schedules, LA-NSCLC treated with concurrent chemo-radiotherapy is characterized by a poor 2-year overall and progression-free survival, typically <60 and 30% respectively, with median OS ranging between 2 and 2.5 years, even in the most recent randomized series (45, 46). Analyses of failure patterns after CRT reveal a substantial contribution of locoregional recurrence, but an even greater proportion of about 50% of patients experiencing distant progression (47). Thus, it seems neither RT's qualities in terms of local control, nor the systemic antitumor effects of chemotherapy are sufficient to offer long-term disease control in all LA-NSCLC patients. Therefore, this setting may represent an exciting opportunity for the development of innovative strategies integrating immunotherapeutic agents into combined modality treatment. This is why it is not surprising that LA-NSCLC was the first clinical entity in lung cancer in which the concept of immune modulation with RT was explored.

#### Available Clinical Evidence (Table 1)

The earliest clinical data on IT-RT combinations in NSCLC date back to the 1970's and 80's, when immuno-oncology was still in its infancy. At the time, experience in leveraging the immune system in order to eradicate cancer cells was mostly limited to the use of non-specific immunostimulants.

**Table 1:** Clinical studies evaluating immunotherapy-radiotherapy combinations in locally advanced non-small cell lung cancer (LA-NSCLC), with primary endpoint results published or presented during the last decade (2009–2019)^a^.

**References**	**Phase**	**NSCLC setting (accrual)**	**Immunotherapy**	**Radiotherapy**	**Design**	**Primary outcome**
**ANTIGEN-SPECIFIC IMMUNOTHERAPY**						
Ohyanagi et al. (48)	I	Stage III, unresectable, CR/PR/SD after CRT (*N* = 6)	Tecemotide	≥50 Gy, sequentially or concurrently with CT	CRT > tecemotide^b^	≥1 AE in 83.3% of pts, all G1
Butts et al. (49)	IIB	Stage IIIB, CR/PR/SD after CRT (*N* = 65)^c^	Tecemotide	Dose NS, sequentially or concurrently with CT	• CRT > BSC + tecemotide^b^ • CRT > BSC	Median OS 30.6 vs. 13.3 m (HR 0.548, 95% CI 0.301–0.999)^c^
Mitchell et al. (50) (START)	III	Stage III, unresectable, CR/PR/SD after CRT (*N* = 1,239)	Tecemotide	≥50 Gy, sequentially or concurrently with CT	• CRT > tecemotide^b^ • CRT > placebo	Median OS 58.7 vs. 57.3 m (HR 0.89; *p* = 0.111)
Patel et al. (51)	II	Stage III, unresectable, non-squamous (*N* = 33)	Tecemotide	66 Gy/33 fx, concurrently with CT	CRT > CT > tecemotide + bevacizumab	≥G3 toxicity in 11 pts, G3 hypertension (*n* = 6)
Brunsvig et al. (52)	II	Stage III, inoperable (*N* = 23)	GV1001 + GM-CSF	60 Gy/30 fx, concurrently with CT	CRT > GV1001 + GM-CSF	No treatment-related SAE
Pujol et al. (53)	I/II	Stage III, unresectable, MAGE A3-positive (*N* = 12)^c^	MAGE-A3 immunotherapeutic	NS	CT > RT > MAGE-A3	Treatment-related AE in 7/12 pts; all < G3. Induced CD4+ and CD8+ T-cell response in 5/6 and 2/6 pts resp.^c^
**IMMUNE CHECKPOINT BLOCKADE**						
Antonia et al. (31) (PACIFIC)	III	Stage III, unresectable (*N* = 713)	Durva	54–66 Gy, concurrently with CT	• CRT > durva • CRT > placebo	Median OS NR vs. 28.7 m (HR 0.68; *p* = 0.0025); median PFS 17.2 vs. 5.6 m (HR 0.51)
Durm et al. (54)	II	Stage III, unresectable, CR/PR/SD after CRT (N = 92)	Pembro	59–66.6 Gy, concurrently with CT	CRT > pembro	Median TMDD 22.4 m (95% CI 17.9-NR)
Lin et al. (55) (DETERRED)	II	Stage III, unresectable (*N* = 40)	Atezo	60–66 Gy/30–33 fx, concurrently with CT	• CRT > CT + atezo • CRT + atezo > CT + atezo	≥G3 atezo-related toxicity in 6 pts; G5 TE fistula (*n* = 1). G3 radiation pneumonitis (*n* = 1)
Peters et al. (56, 57) (NICOLAS)	IA/II	Stage III, unresectable (*N* = 79)	Nivo	• 66 Gy/33 fx, concurrently with CT • 66 Gy/24 fx, sequentially after CT	CRT + nivo > nivo	No ≥G3 post-RT pneumonitis, 1-year PFS 50%

*AE, adverse event(s); atezo, atezolizumab; BSC, best supportive care; CI, confidence interval; CR, complete response; CRT, chemo-radiotherapy; CT, chemotherapy; durva, durvalumab; fx, fraction(s); G, grade; GM-CSF, granulocyte-macrophage colony-stimulating factor; Gy, Gray; HR, hazard ratio; m, month(s); nivo, nivolumab; NR, not reached; NS, not specified; pembro, pembrolizumab; PD, progressive disease; PR, partial response; pts, patients; resp., respectively; RT, radiotherapy; SAE, serious adverse event(s); SD, stable disease; surg, surgery; TE, tracheoesophageal; TMDD, time to metastatic disease or death; +, concurrently with; >, followed by*.

a *Included studies published before 2009 are not represented, as the authors feel the quality of these reports may not correspond to the current standards of evidence and/or practice (e.g., due to the use of outdated RT techniques), thus may confound interpretation of the table contents*.

b *Administration of tecemotide was preceded by a single low dose of cyclophosphamide*.

c *For the purpose of this review, only data relevant to the combination of radiotherapy and immunotherapy for LA-NSCLC are represented in this table*.

One in particular, BCG, had garnered substantial interest due to its encouraging results in hematologic malignancies, which soon inspired the development of several pre-clinical experiments and clinical studies in various other cancer types (58). Specifically in lung cancer, trials evaluating BCG or its methanol extraction residue in combination with (chemo)radiation in LA-NSCLC unfortunately failed to show any benefit in terms of survival (59–62). An interesting finding however, was that when administered during or after RT, these drugs seemed to inhibit the development of distant metastasis (59, 60), suggesting that their potential immunogenic effects may be more pronounced in the context of minimal (residual) disease.

The same allegedly applied to levamisole, a drug used as an anthelmintic before its immunotropic properties were discovered in animal cancer models. Several trials investigated levamisole in conjunction with (chemo)radiation, among which a phase III study initiated by the Radiation Therapy Oncology Group (RTOG), unfortunately showing no difference in OS and PFS compared to placebo, in both resectable and unresectable NSCLC (63–67).

Meanwhile in Japan, research efforts were aimed at combining RT with other non-specific immunopotentiators, namely Picibanil (or OK-432) and polysaccharide K (PSK or Krestin). In their non-randomized trial, Ogawa et al. demonstrated an increased survival associated with the use of OK-432 and/or PSK in addition to chemo-radiotherapy, but did not report significance (68).

A decade later, the first clinical data on experimental RT regimens integrating interferons as radiosensitizers in LA-NSCLC started to emerge. Two smaller studies showed that combining natural alpha-interferon or human recombinant IFNɤ with hyperfractionated RT (60 Gy in 1.25–1.5 Gy fractions, twice-daily) resulted in a marked increase in severe treatment-related toxicity (i.e., esophagitis, radiation pneumonitis, and fibrosis) (69, 70). While initial phase I/II results of concurrent recombinant beta-interferon and conventionally fractionated RT (60 Gy in 2 Gy fractions) were more encouraging, a phase III study initiated by the RTOG demonstrated greater rates of both acute and late side effects and failed to confirm any improvement in overall survival (71). Although interferons play a vital role in *in-situ* vaccination, it seems increasing systemic exposure during RT may overstimulate these pathways, thereby inducing radiation damage where it is least needed. Whether this effect could be mitigated if RT doses were delivered more precisely and conformally is an interesting hypothesis but remains unproven.

A final member of the non-specific immunomodulatory drug family, referred to as bovine dialyzable leukocyte extract, was evaluated concomitantly with chemoradiation in a phase I study, demonstrating its safety and ability to increase certain T-cell subpopulations (72).

As described above, RT and vaccine therapy may also offer the potential of synergistic effects. One of the most extensively studied ASI in LA-NSCLC is tecemotide (or L-BLP25). As maintenance treatment after chemoradiation for stage III disease, the anti-MUC1 vaccine first demonstrated an acceptable safety and tolerability profile (48). Subsequent phase II results were promising, thus warranting initiation of one of the largest phase III IT-RT studies to date (49). The START trial enrolled over 1,500 patients and randomly assigned them two to one, to receive either tecemotide or placebo within 4–12 weeks after completion of chemo-radiotherapy, with the primary aim to improve OS (73). Though this primary endpoint was not met overall, the investigators found that patients receiving tecemotide after concurrent chemoradiation did show a significant increase in OS of about 10 months (HR 0.78, *p* = 0.016), as opposed to those who had received sequential chemo-radiotherapy (HR 1.12, *p* = 0.38). This was confirmed in the updated results published a few years later by Mitchell et al., adding exploratory biomarker analyses which demonstrated a potential positive predictive value of high blood levels of soluble MUC1 (sMUC1) for tecemotide therapy (50). Given that negative prognostic associations were observed between high sMUC1 and OS in the placebo group, researchers proposed that an abundance of sMUC1 in the circulation may reflect increased MUC1 expression by tumors and therefore indicate a target for tecemotide-induced T-cell immunity. A subsequent phase III trial, narrowing its study population to concurrent chemoradiation, attempted to confirm the previously demonstrated OS benefit of adjuvant tecemotide but was terminated prematurely due to the discontinuation of the tecemotide program in NSCLC.

More recently, Patel et al. hypothesized that vascular endothelial growth factor-inhibitor bevacizumab may provide additional immunomodulatory benefits when combined with tecemotide following definitive chemoradiation for LA-NSCLC (51). Their phase I study met its safety endpoint, but the authors did report grade 4 (1/70) and 5 (1/70) toxicity during maintenance treatment.

A second peptide-based vaccine studied in LA-NSCLC, called GV1001, is derived from the functional domain of human telomerase reverse transcriptase. Overexpression of this enzyme in cancer cells helps maintain the integrity of telomere sequences, thereby allowing them to avoid senescence. In their phase II trial of maintenance vaccination following radiotherapy and docetaxel for stage III NSCLC, Brunsvig et al. demonstrated a trend toward improved PFS in patients exhibiting a GV1001-specific T-cell response (52). The benefits of GV1001 in the context of radiation may not be limited to immune activation, as they may also include antifibrotic effects (74).

The cancer/testis antigen, MAGE-A3, has also been proposed as a target for ASI in NSCLC and was investigated in one phase I trial (53). One of four cohorts (*n* = 12) integrated RT into protocol treatment, adding the MAGE-A3 immunotherapeutic after sequential chemoradiation in unresectable stage III NSCLC. In addition to demonstrating an acceptable toxicity profile, a higher prevalence and magnitude of MAGE-A3-specific T-cell responses were observed following chemoradiation in comparison to other study arms not incorporating RT, suggesting irradiated tumor tissue may present an ideal substrate for this particular vaccine.

Several reports, originating primarily from Asian countries, have described therapeutic efficacy of combining chemoradiation with adoptive immunotherapy. A meta-analysis by Qian et al. concluded that the addition of immune cell reinfusion (i.e., DC-CIK, CIK, LAK, or TIL) to conventional NSCLC treatments significantly improved 2-year OS (OR 2.45, 1.60–3.75; *p* < 0.001) (75). Even more recently, a second meta-analysis focusing on CIK treatment in lung cancer demonstrated increased objective response and disease control rates, with 1- and 2-year OS in favor of combined IT-RT treatment in the NSCLC subgroup analyses (76).

Whereas, the previously discussed immunotherapeutics are either no longer under investigation in conjunction with RT for LA-NSCLC, or still in experimental stages of development, a true paradigm shift in the treatment of LA-NSCLC was brought about by the introduction of ICB. In this context, most studies to date have investigated RT in conjunction with monoclonal antibodies targeting the PD(L)-1 receptor interaction. First, consolidation pembrolizumab (anti-PD-1) after chemoradiation in patients with unresectable stage III NSCLC demonstrated increased 2-year PFS (44.6%) and time to metastatic disease or death (TMDD; median 22.4 months) compared to historical controls (54), but the most convincing results to date were achieved with PD-L1 inhibition. The PACIFIC study, a randomized phase III trial evaluating durvalumab or placebo after concurrent chemo-radiotherapy for unresectable stage III disease, initially achieved very convincing results in terms of PFS (HR 0.52) and TMDD (median 23.2 months, HR 0.52) (77). More recently, the trial also showed practice-changing evidence through a significantly prolonged OS of maintenance durvalumab as compared with placebo (HR 0.68, *p* = 0.0025) across all prespecified subgroups (31). This subsequently led to its FDA and EMA approval as standard treatment for tumors with a PD-L1 expression of ≥1% in the majority of jurisdictions. In addition, although there is currently no formal evidence on the effect of durvalumab following sequential chemo-radiotherapy in LA-NSCLC, some countries have already granted approval for this setting as well. Nevertheless, further research is ongoing to define the impact of durvalumab after sequential chemoradiation, as it is recognized that results may not simply be transferable due to aspects inherent to the treatment approach as such and/or because of a difference in patient population, as clearly demonstrated by the START trial. Moreover, since detailed data of the RT delivered in the PACIFIC study, such as dose-volume parameters, are lacking, more thorough RT quality assurance will be of utmost importance in future clinical trials.

The proof of durvalumab's manageable safety profile as a sequential treatment after chemoradiation, in line with previous studies of ICB monotherapy, encouraged researchers to attempt combining all three treatment modalities concurrently. In the DETERRED trial, 40 LA-NSCLC patients were randomized 3:1 to receive standard chemo-radiotherapy with or without concomitant atezolizumab (anti-PD-L1) followed by atezolizumab maintenance treatment (55). No increased toxicity was found when comparing both regimens. Similarly, the recently published interim safety analysis of the ETOP NICOLAS study (*n* = 21) established that the addition of nivolumab (anti-PD-1) concurrently to chemoradiation followed by nivolumab maintenance, did not lead to the occurrence of grade ≥3 pneumonitis by 3 months post-RT (56). Regrettably, phase II results of this trial presented at the 2019 European Society of Medical Oncology congress showed it did not meet its primary efficacy endpoint with a 1-year PFS of 50% (57). Further evidence from other ongoing trials (**Table 3**) are eagerly awaited.

### Metastatic NSCLC

About half of all lung cancer patients are diagnosed with advanced disease (44). Though the therapeutic landscape of metastatic NSCLC (M-NSCLC) has changed tremendously over the past decade, 5-year relative survival remains low at around 6%. Traditionally, RT is offered as a palliative treatment option for these patients (78). In recent years, however, there is growing evidence that RT could also be offered as a local—potentially curative—treatment in a subset of M-NSCLC patients who present with a limited number of metastases, termed oligometastatic disease (79). In this context, stereotactic body radiotherapy (SBRT), using high doses per fraction and precise dose delivery, is often proposed to ablate visible lesions, built upon the finding that further disease progression most often originates from these known disease sites (80). Recent randomized controlled trials (RCTs) have confirmed this hypothesis, the ablative approach yielding OS and PFS benefit (81–83). Nevertheless, this evidence was gathered at a time when IT had not yet made its way into standard first- and/or second-line treatment for M-NSCLC. Considering the potential immunogenicity of RT, perhaps its benefits could extend beyond the oligometastatic setting, boosting immunotherapeutic efficacy through *in-situ* vaccination and the induction of abscopal responses, while debulking sites of gross disease in polymetastatic patients. IT, in turn, may reduce tumor loads or slow down the process of metastatic spread, creating a window of opportunity for local ablative treatments, such as SBRT.

#### Available Clinical Evidence (Table 2)

As in LA-NSCLC, research efforts into IT-RT combinations for the treatment of M-NSCLC date back to a time when ICB had not yet been developed. Studies with limited patient numbers have explored the potential benefit of different IT-RT combinations.

**Table 2:** Clinical studies evaluating immunotherapy-radiotherapy combinations in metastatic non-small cell lung cancer (M-NSCLC), with primary endpoint results published or presented during the last decade (2009–2019).

**References**	**Phase**	**NSCLC setting (accrual)**	**Immunotherapy**	**Radiotherapy**	**Design**	**Primary outcome**
**NON-SPECIFIC IMMUNOTHERAPY**						
van den Heuvel et al. (84)	IB	Stage IV, CR/PR/SD after 1st line CT (N = 13)	NHS-IL2	20 Gy/5 fx, single pulmonary nodule	RT > NHS-IL2	≥G3 treatment-related toxicity in 3 pts
Golden et al. (85)	NS	Stage IV, ≥3 sites of measurable disease, SD/PD on CT (*N* = 18)^a^	GM-CSF	35 Gy/10 fx, 2 lesions consecutively	CT + RT lesion 1 + GM-CSF > CT + RT lesion 2 + GM-CSF	Abscopal response in 4/18 pts
Ohri et al. (86)	II	Stage IV, ≥2 measurable disease sites (*N* = 9)	CDX-301	30–54 Gy/1–5 fx, single intrathoracic site of disease	SBRT + CDX-301	5/9 pts with PFS at 4 m
**ANTIGEN-SPECIFIC IMMUNOTHERAPY**						
Papachristofilou et al. (87)	IB	Stage IV, PR/SD after 1st line CT or TKI, ≥2 sites of disease (*N* = 26)	CV9202	20 Gy/4 fx, single lesion	• RT + CT + CV9202 • RT + CV9202 • RT + TKI + CV9202	≥G3 treatment-related AE in 4/26 pts
**IMMUNE CHECKPOINT BLOCKADE**						
Formenti et al. (88)	I/II	Stage IV, ≥2 measurable metastatic sites (*N* = 39)	Ipi	• 30 Gy/5 fx • 27 Gy/3 fx Single lesion	RT + ipi	CR, PR and SD in 2, 5 and 5/21 evaluable pts resp.
Tang et al. (89)	I	Stage IV, ≥2 sites of disease (*N* = 21)	Pembro	• 50 Gy/4 fx, single liver or lung lesion • 45 Gy/15 fx, SIB allowed up to 60 Gy larger field	RT + pembro	G2 and G3 treatment-related AE in 8 and 3/21 pts resp.
Kumar et al. (90) (PEAR)	I	Stage IV, requiring palliative thoracic RT (*N* = 14)	Pembro	• 20 Gy/5 fx • 36 Gy/12 fx	RT + pembro	No DLT
Decker et al. (91)	I/II	Stage IV, ≥2 measurable disease sites (*N* = 8)	Pembro	30 Gy/3–5 fx, single site of disease	Pembro until irPD > SBRT + pembro	No ≥G2 treatment-related AE during and post-SBRT
Moreno et al. (92)	I	Stage IV, PD after ≥1st line treatment, requiring palliative RT (*N* = 53)	Cemi	27 Gy/3 fx	• RT + cemi • Cemi	G5 treatment-related pneumonitis (*n* = 1). ORR 18.2 vs. 40.0%; DCR 72.7 vs. 60/0%
Alameddine et al. (93)	I	Stage IV, ≤ 10 cc untreated brain metastases (*N* = 7)^a^	Nivo	15–20 Gy/1 fx, brain metastasis	SRS + nivo	Treatment-related AE in 3/5 evaluable pts
Miyamoto et al. (94)	NS	Stage IV, ≥1 lesion amenable to SBRT outside brain/bone (*N* = 6)	Nivo	25.5–48 Gy/3–4 fx, single lesion	SBRT > nivo	G3 pneumonitis in 1/6 pts
Theelen et al. (95) (PEMBRO-RT)	II	Stage IV, ≥2 separate lesions, after ≥1st line treatment (*N* = 76)	Pembro	24 Gy/3 fx, single tumor site	• SBRT > pembro • Pembro	ORR at 12 w 36 vs. 18% (*p* = 0.07)
Luke et al. (96)	I	Stage IV, ≥2 metastases, after ≥1st line treatment (*N* = 7)^a^	Pembro	30–50 Gy/3–5 fx, 2–4 metastases, partial for metastases >65 mL	SBRT > pembro	≥G3 treatment-related toxicity in 6/73 pts
Bauml et al. (97)	II	Stage IV, ≤ 4 metastases (*N* = 45)	Pembro	Stereotactic or standard fraction, dose NS	LAT > pembro	PFS after LAT 19.1 m vs. historical 6.6 m (*p* = 0.005)

*AE, adverse event(s); atezo, atezolizumab; cc, cubic centimeter; cemi, cemiplimab; CR, complete response; CT, chemotherapy; DCR, disease control rate; fx, fraction(s); G, grade; GM-CSF, granulocyte-macrophage colony-stimulating factor; Gy, Gray; m, month(s); mL, milliliter; ipi, ipilimumab; ir, according to immune-related response evaluation criteria in solid tumors; LAT, local ablative treatment (i.e. surgery, chemotherapy, radiotherapy, radiofrequency ablation or a combination of the above); nivo, nivolumab; NR, not reached; NS, not specified; ORR, objective response rate; pembro, pembrolizumab; PD, progressive disease; PR, partial response; pts, patients; resp., respectively; RT, radiotherapy; SAE, serious adverse event(s); SD, stable disease; surg, surgery; TE, tracheoesophageal; TKI, tyrosine kinase inhibitor; TMDD, time to metastatic disease or death; +, concurrently with; >, followed by*.

a *For the purpose of this review, only data relevant to the combination of radiotherapy and immunotherapy for M-NSCLC are represented in this table*.

Among the first experiments with RT and immunocytokine therapy in lung cancer demonstrated an acceptable safety profile and potential immunogenicity of Selectikine or NHS-IL2 (genetically modified interleukin-2 fused with DNA-targeting antibody NHS76), administered after local irradiation of a single pulmonary lesion in 13 M-NSCLC patients (84). Interestingly, both long-term survivors developed thyroiditis during treatment, which was only seen in one other patient, indicating that the incidence of immune-related adverse events may be linked to intrinsic IT susceptibility.

Recognizing the importance of DCs in RT-induced immune activation, Golden et al. attempted to stimulate DC maturation by combining radiation with granulocyte-macrophage colony-stimulating factor (GM-CSF), ultimately aiming to elicit abscopal responses (85). Almost half of the 41 patients studied had M-NSCLC (*n* = 18), four of whom demonstrated a partial (2/4) or complete (2/4) response in non-irradiated lesions. In addition, patients experiencing an abscopal response (11 in total) showed significantly better OS (HR 2.06).

Similar to GM-CSF, the feline McDonough sarcoma-like tyrosine kinase 3 ligand CDX-301 has the ability to induce differentiation of bone marrow precursor cells into distinct subsets of DCs. Preliminary results from an ongoing trial investigating SBRT to an intrathoracic disease site and concurrent CDX-301 in M-NSCLC are promising, with more than half of patients (5/9) achieving partial remission (86). Of note, all five responders had been previously treated with ICB.

Though disappointing phase III results, such as those of the START trial in LA-NSCLC (50, 73) and the STOP trial investigating maintenance belagenpumatucel-L in M-NSCLC (98), might have negatively impacted general enthusiasm for antigen-specific immunotherapeutic approaches in NSCLC, novel mRNA-based vaccines may be more promising. Targeting five and six different TAAs respectively, CV9201 and its successor CV9202 have shown an acceptable safety profile and evidence of immune activation when delivered after or concurrently with local fractionated RT for stage IIIB-IV disease (87, 99). Additional research is required to determine whether this combination treatment can improve long-term outcome.

The largest body of available IT-RT evidence in M-NSCLC describes the marriage of radiation and ICB. While Formenti et al. performed a pioneering study on abscopal effects with ipilimumab (88), research subsequently published focused mainly on combining RT with anti-PD-1 antibodies, due to their proven efficacy as monotherapy in M-NSCLC.

Early results of two phase I trials, comparing different—radical or palliative—RT dose-fractionation schedules given concurrently with escalating doses of pembrolizumab, demonstrated acceptable toxicity of combined treatment and encouraging disease control rates (DCR) of up to 68% (89, 90). Moreover, Kumar et al. observed a trend for improved PFS with higher RT doses, supporting the notion that these could potentially be more immunogenic (90). Another cleverly designed study attempted to elucidate the role of radiation as a salvage treatment for M-NSCLC refractory to ICB, adding SBRT onto pembrolizumab treatment only at the time of disease progression (100). Stable disease and systemic responses were achieved in 10 and 2 of the 21 patients who completed study treatment, respectively.

Early safety data on the concurrent use of other anti-PD-1 drugs (e.g., cemiplimab) with RT appear to be reassuring (92), although caution is advised when treating brain metastases radiosurgically in conjunction with nivolumab, especially in the presence of significant peritumoral edema (93).

Sequential approaches were also evaluated, initially for nivolumab (94), but more recent phase I and II trials focused on pembrolizumab after local ablative treatment for M-NSCLC. For instance, Theelen et al. performed a RCT of pembrolizumab either without or after SBRT (3 × 8 Gy) of a single NSCLC metastasis (95). While the study's primary endpoint criteria were not met, a significant improvement of DCR was observed in the experimental arm (64 vs. 40%; *p* = 0.04). Moreover, subgroup analyses showed patients benefiting most from SBRT were those with PD-L1 negative tumors at baseline. This finding is particularly intriguing, since this is a population for which single-agent PD-(L)1 inhibition is known to be of limited benefit. Perhaps, pembrolizumab following SBRT may represent a less toxic alternative to chemoimmunotherapy when aiming to enhance response rates in M-NSCLC patients with a low PD-L1 tumor proportion score.

Despite promising results, some authors have advocated abandoning the single-site abscopal approach and instead propose to irradiate as much of the tumor burden as can be safely achieved (101). They hypothesize that targeting only one lesion may fail to account for tumor heterogeneity and immunosuppressive features of bulky disease, thereby limiting the probability of RT-induced systemic antitumor immune activation. Luke et al. applied this logic in their phase I study, allowing multisite SBRT of 2–4 metastases up to 1 week before starting pembrolizumab in patients with advanced solid tumors (96). Translating this concept into the oligometastatic NSCLC setting, Bauml et al. initiated a phase II trial offering adjuvant pembrolizumab to patients with a limited tumor burden (≤4 metastases) after eradication of all known sites of disease (97). Median PFS exceeded that of historical controls, but whether this benefit could be explained by a potential IT-RT synergy remains unclear, as subgroup analyses comparing irradiated to radiation-naive patients could not be performed. Questions like these should be answered in RCTs, preferably stratifying patients according to tumor burden, such as the ongoing IMMUNOSABR2 study. Other ongoing IT-RT trials in M-NSCLC are shown in Table 3.

**Table 3:** Currently ongoing trials (i.e. not yet recruiting, recruiting and enrolling by invitation) evaluating immunotherapy-radiotherapy combinations in non-small cell lung cancer^a^.

**Study identifier (acronym)**	**Phase**	**NSCLC setting (accrual)**	**Immunotherapy**	**Radiotherapy**	**Design**	**Outcome**	**Institution/group**
NCT02599454	I	Stage I, inoperable (*N* = 33)	Atezo	50 Gy/4–5 fx	SBRT + atezo	MTD (DFS, ORR)	University of California, Davis
NCT03148327 (ISABR)	I/II	Stage I/IIA, inoperable (*N* = 105)	Durva	• 50 Gy/4 fx • 54 Gy/3 fx • 65 Gy/10 fx	• SBRT + durva • SBRT	Tox, PFS (OS, LC)	Jonsson Comprehensive Cancer Center
NCT03446547 (ASTEROID)	II	Stage I-IIA, not suitable for surg (*N* = 216)	Durva	3–4 fx, dose NS	• SBRT > durva • SBRT	PFS (OS, LC, QoL)	Vastra Gotaland Region
NCT03833154 (PACIFIC-4)	III	Stage I-II lymph node negative, planned for SBRT (*N* = 630)	Durva	NS	• SBRT > durva • SBRT > placebo	PFS (OS, QoL, tox, IM)	AstraZeneca
NCT03110978	II	Stage I-IIA or isolated lung parenchymal recurrent/persistent (*N* = 1 40)	Nivo	• 50 Gy/4 fx • 70 Gy/10 fx	• SABR + nivo • SABR	EFS (OS, tox)	M.D. Anderson Cancer Center
NCT03574220	I	Stage IA-IIB, inoperable (*N* = 15)	Pembro	• 50 Gy/5 fx • 60 Gy/3 fx	SBRT > pembro	Tox (DMFS, DFS, OS, LC)	Case Comprehensive Cancer Center
NCT03383302 (STILE)	Ib/II	Stage I-IIA, not suitable for surg (*N* = 31)	Nivo	• 54 Gy/3 fx • 55 Gy/5 fx	SBRT > nivo	Tox (DFS, OS, QoL, IM)	Royal Marsden NHS Foundation Trust
NCT03546829	I	Early-stage, planned for SBRT (*N* = 40)	Vancomycin	NS	• SBRT > vancomycin • Vancomycin > SBRT	IM	Abramson Cancer Center of the University of Pennsylvania
NCT01720836	I/II	Stage IA-IIIB (*N* = 30)	Hiltonol (MUC1 + poly-ICLC)	NS	SOC > Hiltonol	IM	University of Pittsburgh Medical Center
NCT03217071 (PembroX)	II	Stage I-IIIA (*N* = 40)	Pembro	12 Gy/1 fx, 50% of primary tumor	• Pembro > SBRT > surg • Pembro > surg	IM (OS, DFS, tox)	University of California, San Francisco
NCT03801902 (ARCHON-1)	I	Stage II-III, unresectable or inoperable (*N* = 24)	Durva	• 60 Gy/15 fx • 60 Gy/30 fx	• Accelerated RT + durva • Conventional RT + durva	Tox (feas, PFS, IM)	NRG Oncology
NCT02621398	I	Stage II inoperable or stage III (*N* = 30)	Pembro	3D-RT or IMRT, 30 fx, dose NS	CRT + pembro	MTD, DLT (ORR, LC, DMFS OS, PFS)	Rutgers Cancer Institute of New Jersey
NCT04013542	I	Stage II unresectable or stage III (*N* = 20)	• Nivo • Ipi	6–7 w, dose NS	RT + nivo + ipi > nivo	Tox (PFS, OS, LC, ORR, DOR)	M.D. Anderson Cancer Center
NCT03523702 (SPRINT)	II	Stage II unresectable or stage III (*N* = 63)	Pembro	4–7 w, dose NS	• PD-L1 <50%: CT + RT • PD-L1 ≥50%: Pembro + RT	PFS (DMFS, OS)	Albert Einstein College of Medicine
NCT04062708 (CHIO3)	II	Stage III, resectable (*N* = 55)	Durva	54 Gy, number of fx NS	CT + durva > surg > RT > durva	Nodal response (pathologic and radiologic ORR, EFS, OS, tox)	Alliance Foundation Trials, LLC
NCT03237377	II	Stage III, resectable (*N* = 32)	• Durva • Treme	45 Gy/25 fx	• RT + durva > surg • RT + durva + treme > surg	Tox, feas (pathologic and radiologic ORR, DOR, OS)	Sidney Kimmel Comprehensive Cancer Center at Johns Hopkins
NCT03871153	II	Stage III, resectable (N2) (*N* = 25)	Durva	45–61.2 Gy/25–34 fx	CT + durva > RT + durva > surg > durva	Pathologic CR (nodal response, tox, PFS)	Indiana University School of Medicine
NCT03631784 (KEYNOTE-799)	II	Stage III, unresectable, 1st line (*N* = 216)	Pembro	60 Gy/30 fx	CRT + pembro > pembro	Tox, ORR (PFS, OS)	Merck Sharp & Dohme Corp.
NCT03663166	I/II	Stage III, unresectable (*N* = 50)	• Nivo • Ipi	60 Gy/30 fx	CRT + ipi > nivo	Tox, PFS (DMFS, ORR)	H. Lee Moffitt Cancer Center and Research Institute
NCT03285321	II	Stage IIIA/B, unresectable or inoperable, CR/PR/SD with CRT (N = 108)	• Nivo • Ipi	59.4–66.6 Gy, number of fx NS	• CRT > nivo • CRT > nivo + ipi	PFS (OS, DMFS, tox)	Big Ten Cancer Research Consortium
NCT03589547	II	Stage III, PR/SD with CRT (*N* = 25)	Durva	20 Gy/2–3 fx, primary tumor only	SBRT + durva	Tox, PFS (OS, LC, DMFS)	Brown University
NCT03102242	II	Stage IIIA/B, unresectable (*N* = 63)	Atezo	60 Gy/30 fx	Atezo > CRT	DCR	Alliance Foundation Trials
NCT03644823 (COM-IT-1)	II	Stage III-IV, palliative treated (*N* = 30)	Atezo	18 Gy/3 fx	RT + atezo	Tox (PFS)	Oslo University Hospital
NCT03774732 (NIRVANA- Lung)	III	Stage IIIB-IV (*N* = 510)	• Nivo • Pembro • Atezo	• SABR: NS • 3D-RT: 18 Gy/3 fx	• RT + ICB • ICB	OS (ORR, PFS, LC, QoL, tox)	UNICANCER
NCT02839265	II	Stage III-IV, ≥2 measurable disease sites (*N* = 29)	CDX-301	30-54 Gy/1–5 fx, single intrathoracic site of disease	SBRT + CDX-301	PFS (DLT)	Albert Einstein College of Medicine
NCT03965468 (CHESS)	II	Stage IV, oligometastatic (≤3 lesions) (*N* = 47)	Durva	• SBRT: up to 10 fx, dose NS • Definitive RT: 60–66 Gy, fx NS	SBRT + CT + durva > surg or definitive RT + durva	PFS (OS, ORR, DOR, QoL, tox)	European Thoracic Oncology Platform
NCT03275597	Ib	Stage IV, oligometastatic (≤6 lesions) (*N* = 21)	• Durva • Treme	30–50 Gy/5 fx, all sites of disease	SBRT > durva + treme	Tox (PFS, OS, IM)	University of Wisconsin, Madison
NCT03509584	I	Stage IV (*N* = 24)	• Nivo • Ipi	24 Gy/3 fx, single bone or extracranial metastasis	• RT + nivo • RT + nivo + ipi	Tox	Assistance Publique Hopitaux De Marseille
NCT03223155 (COSINR)	I	Stage IV (*N* = 80)	• Nivo • Ipi	3–5 fx, dose NS, 2–4 sites	SBRT > nivo + ipi	Tox (ORR, LC, IM)	University of Chicago
NCT03168464	I/II	Stage IV, ≥2 measurable metastatic sites (*N* = 45)	• Nivo • Ipi	30 Gy/5 fx, single lesion	RT + ipi > nivo + ipi	ORR (PFS, DOR, OS, IM)	Weill Medical College of Cornell University
NCT02444741	I/II	Stage IV, ≥2 disease sites (*N* = 104)	Pembro	• 4 fx: SBRT • 15 fx: IMRT, 3D-RT or PBRT	• Pembro + RT • Pembro > RT upon PD	Tox, ORR (PFS, OS)	M.D. Anderson Cancer Center
NCT03035890	NS	Stage IV, ≥3 disease sites (*N* = 33)	• Nivo • Pembro • Atezo	• 24–45 Gy/3 fx • 30–50 Gy/5 fx Single lesion	RT + ICB	ORR (PFS, OS, tox, QoL)	West Virginia University
NCT03825510	NS	Stage IV, ≥2 lesions amenable to SBRT (*N* = 100)	• Nivo • Pembro	3–5 fx, dose NS, ≤ 3 sites	SBRT > ICB	OS, tox (PFS, LC)	Crozer-Keystone Health System
NCT03867175	III	Stage IV, ≤ 8 disease sites (*N* = 116)	Pembro	3–10 fx, dose NS	• SBRT > Pembro • Pembro	PFS (OS, LC, tox)	Wake Forest University Health Sciences
NCT03391869 (LONESTAR)	III	Stage IV (*N* = 270)	• Nivo • Ipi	NS	Nivo + ipi > LCT > nivo + ipi	OS (PFS, tox, QoL)	M.D. Anderson Cancer Center
NCT03705403 (IMMUNOSABR2)	II	Stage IV (*N* = 130)	Darleukin (L19–IL2)	24 Gy/3 fx	• SOC + Darleukin • SOC	PFS (OS, QoL, IM)	Maastricht University
NCT03158883	I	Stage IV, ≥2 measurable disease sites, non-responsive or refractory to ICB (*N* = 26)	Ave	50 Gy/5 fx	SBRT + ave	ORR (OS, PFS, DCR, DOR)	University of California, Davis
NCT03224871	I	Stage IV, ≥2 disease sites, non-responsive or refractory to ICB (*N* = 30)	• Nivo • Pembro • Intralesional IL-2	24 Gy/3 fx, single lesion	RT + ICB > ICB + IL-2	DLT (DFS)	University of California, Davis
NCT03406468	II	Stage IV, refractory to ICB (*N* = 40)	• Nivo • Pembro • Atezo	• 24 Gy/3 fx • 30 Gy/10 fx • 20 Gy/5 fx • 20–24 Gy/1 fx Single lesion	RT + ICB	PFS (LC, tox)	Maastricht University
NCT03176173	II	Stage IV, ≥1 extracranial disease site, after ≥4 w ICB (*N* = 85)	• Nivo • Pembro • Atezo	≤ 10 fx, dose NS	• RT + ICB • ICB	PFS (tox, OS, IM)	Stanford University
NCT03044626 (FORCE)	II	Stage IV, non-squamous, 2nd or 3rd line (*N* = 130)	Nivo	20 Gy/5 fx, single metastatic site	• RT + nivo • Nivo	ORR (PFS, OS, tox, QoL)	AIO-Studien-gGmbH
NCT03489616 (CRAGMOLC)	NS	Stage IV, oligometastatic (2–5 metastases), PR/SD after first-line CT (*N* = 45)	rhGM-CSF	BED >45 Gy, >4 Gy per fx	• CT + RT + rhGM-CSF • CT	PFS (OS)	Shandong Cancer Hospital and Institute

*3D-RT, 3-dimensional conformal radiotherapy; atezo, atezolizumab; ave, avelumab; BED, biologically effective dose; CR, complete response; CRT, chemo-radiotherapy; CT, chemotherapy; DCR, disease control rate; DFS, disease-free survival; DLT, dose-limiting toxicity; DMFS, distant metastasis-free survival; DOR, duration of response; durva, durvalumab; EFS, event-free survival; feas, feasibility; fx, fraction(s); Gy, Gray; ICB, immune checkpoint blockade; IL, interleukin; IM, immunomonitoring; IMRT, intensity-modulated radiotherapy; ipi, ipilimumab; MTD, maximum tolerated dose; nivo, nivolumab; LC, local control; LCT, local consolidation treatment; NS, not specified; NSCLC, non-small cell lung cancer; OS, overall survival; ORR, overall response rate; PBRT, proton beam radiotherapy; PD, progressive disease; pembro, pembrolizumab; PFS, progression-free survival; PR, partial response; QoL, quality of life; rhGM-CSF, human recombinant granulocyte-macrophage colony-stimulating factor; RT, radiotherapy; SABR, stereotactic ablative radiation therapy; SBRT, stereotactic body radiation therapy; SD, stable disease; SOC, standard of care; tox, toxicity; treme, tremelimumab; w, week(s); +, concurrently with; >, followed by*.

a *Only studies focusing exclusively on a NSCLC patient population are represented in this table*.

### Early-Stage NSCLC

Early-stage NSCLC (ES-NSCLC) represents about 15–20% of all new lung cancer diagnoses (44). According to the latest consensus guidelines, surgery remains the treatment of choice for operable ES-NSCLC patients. For those unfit for or unwilling to undergo surgical resection, SBRT is now the gold standard, with an excellent safety profile and local control rates of approximately 90% at 5 years (1). Unfortunately, as in LA-NSCLC, the issue remains distant relapse, occurring in up to 20% or more (102–104). This number rises to over 30% in most reports when regional recurrences are taken into account. Certain clinical and molecular features associated with poor prognosis have been identified (105, 106), justifying possible treatment intensification in a subset of ES-NSCLC patients. In this context, systemic treatment options could be considered in order to eradicate micrometastatic disease and, in doing so, improve long-term outcome. However, as factors determining patients' operability often coincide with those affecting their eligibility for chemotherapy, a unique opportunity presents itself for treatments with a relatively modest toxicity profile, such as IT. Moreover, IT may offer additional benefits in terms of potential synergistic effects with SBRT as detailed above. This rationale is further supported by immune mechanistic studies demonstrating hypofractionated RT for stage I NSCLC may stimulate immune activation (107). As of yet, clinical data of combined IT-RT approaches in ES-NSCLC are lacking, but trials investigating their safety and efficacy are well underway (Table 3).

## Conclusion

As of yet, the lion's share of evidence demonstrating clinical benefit with combined immunotherapy-radiotherapy strategies for NSCLC is situated in locally-advanced disease. Even so, a growing body of pre-clinical and clinical data has allowed further insights into their synergy, providing a strong rationale for extending this potential to other disease entities, such as metastatic NSCLC. In addition, the excellent tolerability profile of many novel immunotherapeutic drugs, both as monotherapy and in conjunction with radiotherapy, as well as technological and technical radiotherapy advances have created a window of opportunity for the development of combined strategies in earlier disease stages. Nevertheless, the success of future trials will require well-reasoned hypotheses for radiotherapy timing, dose and fractionation, in addition to selection of the appropriate partner immunotherapy.

## Author Contributions

MS performed the literature review and search of ongoing clinical trials, on which both authors structured and synthesized the evidence for the manuscript. YL critically revised all the drafts and approved the final version for submission.

### Conflict of Interest

YL reports expert positions for AstraZeneca and RaySearch, outside the current work. The remaining author declares that the research was conducted in the absence of any commercial or financial relationships that could be construed as a potential conflict of interest.
